# Speciation happens in company – not in isolation

**DOI:** 10.1038/s44185-024-00047-5

**Published:** 2024-07-05

**Authors:** Carl Beierkuhnlein

**Affiliations:** 1 Department of Biogeography, University of Bayreuth, Universitaetsstr. 30, 95447 Bayreuth, Germany; 2 Bayreuth Center of Ecology and Environmental Research, BayCEER, University of Bayreuth, Universitaetsstr. 30, 95447 Bayreuth, Germany; 3 Geographical Institute of the University of Bayreuth, GIB, Universitaetsstr. 30, 95447 Bayreuth, Germany; 4 Departamento de Botánica, Universidad de Granada, 18071 Granada, Spain

**Keywords:** Ecology, Evolution, Ecology

## Abstract

Oceanic islands are considered the classic arenas for allopatric speciation and adaptive radiation. Established concepts of speciation and endemism are strongly focused on spatial and temporal scales. However, biotic interactions and ecological drivers, although widely recognized as playing a role, still need to be integrated into our understanding of these processes. Here, I highlight ecosystems as the evolutionary arena within islands. Ecosystem functioning, such as the regulation of abiotic fluxes of energy and matter, has been intensely studied in the context of climate change and biodiversity loss. Biogeography, on the other hand, when it focuses on speciation and endemism, often lacks a functional understanding of the ecosystem beyond species lists. This contribution aims to stimulate a stronger integration of ecological processes, assembly rules, and vegetation structures into future biogeographical and macroecological studies.

## Introduction

Islands serve as model systems and natural experiments that contribute to our understanding of the fundamental processes governing population dynamics, biodiversity, and speciation^1–6^.

**Fig. 1:**
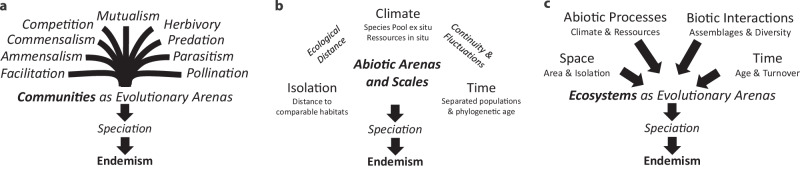
Coenotic, abiotic, and ecological arenas of speciation and endemism. **a** Diversity of biotic interactions supporting and suppressing selection processes and speciation; **b** Abiotic drivers of speciation, spatial isolation of populations, and temporal scales related to life cycles and mutation rates; **c** Integration of abiotic and biotic drivers related to spatial and temporal scales resulting in ecosystems are the evolutionary arenas controlling speciation and endemism on islands.

Speciation is considered to be a function of spatial isolation, climatic conditions, time, biotic interactions, and the availability of ecological niches. Especially the concept of adaptive radiation, and the discussions surrounding it, are closely tied to the field of island biogeography^7–9^. This is why oceanic islands, in particular, are considered “evolutionary arenas”^10^. Due to spatial isolation, speciation is directly linked with endemism on islands. However, despite of spatial and ecological separation of populations, speciation never happens in absolute isolation. Intraspecific interactions promote the selection of best adapted offspring. Specifically, the community and ecosystem matrix constitute the complex biotic framework which facilitates evolution (Fig. 1).

Macroecological evidence for the role of spatio-temporal and climatic drivers is built on big data. Nevertheless, there are phylogenetic patterns in endemic species that cannot be understood with a mere spatial, temporal, or climatic perspective (e.g., laurel pigeons). If the size and the age of islands are not sufficient to explain patterns of speciation, additional processes could be even more effective for diversification. Biotic interactions and respective assembly rules are candidates. One prominent example in oceanic island plants is the role of mycorrhizal fungi^11^. The isolation of oceanic islands may act as a filter for mutualistic and symbiotic fungal partners. However, biotic interactions are likely to increase with the age of ecosystems and differ between various types of ecosystems, too. The influence of this complexity on the selective success or failure of propagules cannot be easily generalized.

The main aim of this contribution is, to highlight the link of the size of ecosystems within islands with plant endemism. This is done at the scope of the Canarian Islands, a well-studied archipelago with repeated occurrences of comparable ecosystems^12^. Currently, approximately 25% of the Canary Island flora is endemic, 35% is considered to be probably native, but non-endemic, and 40% is non-native including some invasive species^12^. Obviously, these species are not distributed equally within islands. Endemism in island ecosystems is driven by site conditions and biotic interactions. The interplay between biotic and abiotic processes is resulting in ecological speciation that differs between ecosystems.

## Spatial scales and filters

Species-Area Relationships (SAR) are a fundamental topic in ecology and biogeography^13–15^. The spatial characteristics of islands, namely their size and distance from continents or other islands, were related to establishment and extinction rates resulting in specific numbers of species in equilibrium^16^. The ambition to translate this theory to isolated continental habitats stimulated further progress in the field of Species-Area Relationships^17^. As a certain degree of variability within a number of species populations is the prerequisite for selection and speciation, evolutionary processes were also linked to SAR^18^. Moody^19^ shows differences in species-area relationships between endemic, native, and exotic plant species. Additionally, endemic richness tends to increase with isolation of entire islands. However, spatial isolation of ecosystems also applies to ecosystems within islands. On mountainous islands, elevational and climatic zonation influence the spatial extent of habitats (decreasing) and ecological isolation (increasing). Accordingly, Steinbauer et al.^20–22^ found a general increase in the proportion of endemic plant species with altitude.

**Fig. 2:**
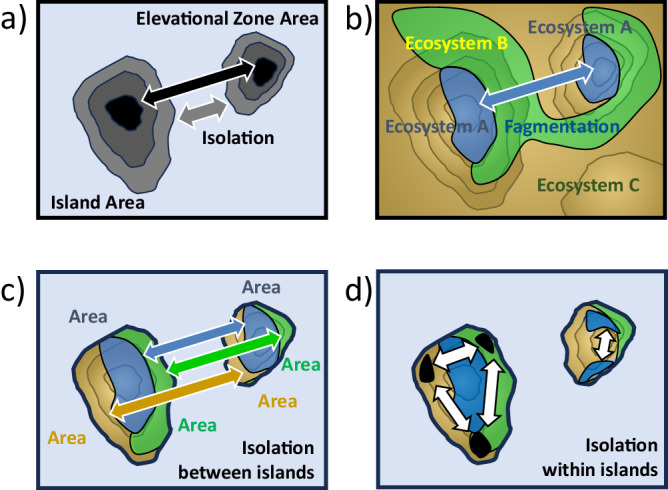
Area and isolation as spatial drivers of speciation. **a** Island biogeography theory is focused on spatial area and isolation, which can also be applied to endemism and elevational zones within islands. **b** In a continental context, ecosystems at mountain tops can be seen as fragmented “sky islands” but lower elevational zones are often connected. **c** Topographically diverse islands, however, exhibit patterns of ecosystems that are more complex than just elevational gradients with different ecosystem area and isolation. **d** Within islands, ecosystems can be fragmented and isolated in various ways.

Although the initial motivation to explain Species-Area Relationships was to translate findings from islands to isolated habitats and ecosystems on continents^16^, it was obvious that these continental habitats and their species experience a different kind of isolation. Ecotones and other transitional zones between patches, embedded into a heterogeneous matrix, result in less distinct spatial limits of isolated ecosystems (Fig. 2). Bedrock and soils, and thus resource availability (water and nutrients), are more diverse in the continental realm. For this reason, islands located on the oceanic crust provide an ideal study system for ecological and evolutionary research because they are primarily formed through identical processes - namely volcanic activities. These islands, while sharing a common genesis, also present a wide range of factors for investigation such as different climatic conditions, sizes, elevations, and ages.

Spatial distance and elevational gradients are linked with ecological isolation of populations. While the process of speciation can indeed be influenced by the extent and length of geographic isolation from ancestor populations, it would be incorrect to assume that this spatial and temporal context alone provides a comprehensive explanation for the current patterns of plant endemism.

Globally, many independent colonization events happened in history and until today^23^. In consequence, dispersal filtering because of pure geographic isolation is unlikely to serve as the only factor for speciation. A variety of biotic interactions and ecological drivers also contribute to the spatio-temporal context of speciation. It is evident that, in addition to the size of islands, the three-dimensional structure as well as the respective heterogeneity of site conditions within islands during their ontogenetic development are important^24–26^. Besides volcanic eruptions, geomorphodynamic processes such as giant landslides and erosion, which are dependent on both, slope energy and climatic conditions, add to the life cycle of islands. However, the phylogeny of island biota is not necessarily linked to the ontogeny of islands^27^.

Many life forms are bound to specific ecosystems, such as laurophyllous trees to the laurel forest on La Palma. For such species, it is the area, elevational range, fragmentation, climate, natural disturbance regime, and anthropogenic impacts of a specific ecosystem, which are decisive for the establishment of populations, not the area and elevation of the island in total. Islands are not homogeneous spatial entities. Endemic species were found to be linked with climatically rare site conditions on the Canary Islands^28^. And climatically rare habitat conditions are likely to be met by specific ecosystems.

Habitat isolation within islands is an important extrinsic barrier for plant populations, promoting the speciation of island plants^29,30^. Nevertheless, modeling studies consider isolation scenarios rather for the level of entire islands related to continents and for habitat availability the entire island area is taken or a certain elevational zone^31^. On topographically pronounced islands, habitats and ecosystems are not only reflecting elevational zones, but also slope, aspect, major wind direction, cloudiness, and other site conditions. The real evolutionary arena, which is the ecosystem matrix, is widely ignored when islands are seen as one entity.

## Temporal scales, equilibrium, and turnover

Temporal scales relate not only to the age of origin and formation of islands, but also to turnover rates of species assemblages that can be assessed in distinct spatial units^32^. One unifying aspect of the Equilibrium Theory of Island Biogeography^16^ and related approaches^33^ is the assumption that over time a certain equilibrium in species richness as well as an equilibrium in ecological or evolutionary processes will be reached^34^. Early on, this notion was challenged^35^, as, for remote oceanic islands in particular, reaching an equilibrium is highly unlikely.

Due to their isolation and small size, often combined with young age, most oceanic islands are species-poor compared to habitats with comparable climatic conditions on the continent. There are hints that species richness is linked with the likelihood of speciation^36^. However, this would not explain the high number of endemic species on these islands. Most oceanic islands host far more endemic species than areas of the same size in similar latitudes or floristic realms on neighboring continents^37^.

On the Canary Islands, the genus *Aeonium* serves as a quintessential illustration of allopatric speciation in an oceanic archipelago, due to its large number of endemic species, subspecies, and accepted hybrids^12^. Endemic members of the genus *Aeonium* differ strongly in their phylogenetic age, which contradicts conventional expectations and existing models^27^. Single island endemics (SIE) of one island within this genus can be both: recently isolated and rather young but also even older than the oldest part of their island, and may have evolved on other islands before. However, the general time span of speciation fits to the model projections of the General Dynamic Theory^24^, where speciation is thought to be enhanced during the first 5 Mya of an oceanic island’s history^25,26^. This timespan corresponds to the active phase of island building volcanism on most oceanic islands. Environmental heterogeneity is seen as the major contribution to the temporal trajectories of oceanic island biodiversity following the General Dynamic Model (GDM)^38^. Abiotic environmental heterogeneity is highest after the end of the active volcanic phase. Previously speciated populations are likely maintained during this peak heterogeneity due to the diversity of habitats. In the following phase of erosion and loss of habitats, extinction processes are becoming more and more prominent over time.

The radiation of *Aeonium* is thought to have also started ca. 5 Mya ago^39^. Surprisingly, the phylogenetic age of some *Aeonium* species has been estimated as older than the single islands on which they are endemic^39^. Because of their age, former large oceanic islands within Palaeo-Macaronesia, which have been completely eroded away and whose remnants are reflected only in submarine guyots^40,41^, are unlikely places of origin because these islands disappeared from the sea surface long time before the speciation of *Aeonium*. However, these guyots may well have served as stepping-stones for dispersal between islands when partly emerged as flat islands during the long periods of lower sea level in the Pleistocene.

Curto et al.^42^ investigated the complex evolutionary history of *Micromeria* on the Canary Islands, showing that populations on young islands were genetically very diverse and that genetic dissimilarity could be explained through isolation by distance. However, the pattern of genetic differentiation of this genus also shows repeated colonization events and hybridization, which can be modified through ecological filters within islands. Stuessy et al.^43^ discuss the contribution of oceanic island age and plant population sizes on genetic diversity. However, these drivers are also linked to the type and extent of ecosystems within islands.

## Climatic and geomorphological drivers of evolution

The role of climatic fluctuations in extant species distribution patterns is again reflected in the genus, *Aeonium*. The only three described continental *Aeonium* species (one in Morocco, and two in East Africa and Yemen) are apparently very young (ca. 150 ka)^39^, even though they are partly very remote from their closest and substantially older relatives on the Canary Island archipelago. This clearly contradicts the assumption that continental species always provide source populations for allopatric speciation on islands. This pattern of older phyla on oceanic islands and younger phyla on continents hints toward much stronger climatic fluctuations during Earth history (e.g. during the Pleistocene) in continental habitats, resulting in more frequent modifications and even replacement of their species pools. Appropriate continental habitats of *Aeonium* species were very likely once connected through mountain ranges and lowland savannas during periods of moist climate in North Africa^44^, enabling the spread of populations across the continent followed by climatic isolation. Hence, past climatic changes and lost habitat connections on continents need to be considered when explaining climatic drivers of island biota endemism today.

Island climate is closely linked to latitude and topography. The latitudinal position of islands is modified over time through plate tectonic movements. During the Neogene, the African continent and the Canary Island archipelago shifted slightly northward^41^. Even stronger, though, was the role of climatic fluctuations during the Pleistocene in causing species extinctions^45^. These external climatic drivers are combined with the internal dynamics of islands such as volcanism, landslides, and erosion. The fact that the nature and structure of oceanic islands is inherently dynamic was already highlighted long before the geological processes of island formation were fully understood^46^. The creation of bedrock and substrate is the prerequisite for terrestrial habitats, where weathering and soil formation under respective climatic conditions then provide nutrient and soil water availability, which is the basis for plant life. Within islands of the Canary Island archipelago, Otto et al.^47^ suggest that Single-Island Endemic plants have originated on geologically older parts of islands. This indicates that the differentiation of landforms and soils, both reflected in ecosystems, is of importance for speciation. In addition to the increasing resource availability through rock weathering that facilitates plant populations, destructive volcanic forces can be selective towards specific plant traits^48,49^.

## Biotic interactions and ecological speciation

Dobzhansky^50^ explicitly made the link between the development of genotypes and their respective ecological niche. Evidently, the realized ecological niche space is controlled by coexisting species. Plant species do not evolve into an empty space without pre-existing biota. Hence, evolutionary processes should not be seen as detached from their ecological setting. Site conditions and local resources (e.g., light regime, nutrient availability, soil texture, porosity, and depth, precipitation regime, and soil water) together with established and developing communities are decisive for the success of establishment and reproduction, and in consequence, for the selection of morphological and functional traits, phenology, and life cycles.

Biotic processes and interactions that drive selective processes of plant traits are manifold, including mutualistic or antagonistic interactions between plants but also with animals, bacteria, and fungi^51^. Many plants are dependent on other species for pollination and dispersal, but also for nutrient uptake via different types of symbiotic mycorrhiza or the provision of nitrogen and other nutrients through microbiota. Plant-plant interactions include the competition for resources, such as light, water or nutrients, but also the release of toxic compounds, synchronized phenology, longevity, and population turnover of co-existing plant species. The timing and location of such supporting or suppressing processes are likely to be quite a stochastic component with respect to evolutionary trends. Disturbance regimes can generate windows of opportunity for reproduction and affect population dynamics in the context of a local species pool, but successional trajectories can be strongly influenced by priority effects^52^. In consequence, such drivers most likely result in more individualistic and less deterministic species assemblages as a basis for speciation and endemism.

Evolutionary processes are dependent on environmental conditions^53^. However, ecological components in selection have been highlighted mostly for reproductive isolation and speciation of animal populations^54–56^.

## Ecosystems and endemism

Generally, it can be assumed that “diversity begets diversity” and that speciation is stimulated by the size of a species pool and its genetic diversity^57^. However, islands are generally species-poor in comparison with continents and their communities as well as biotic interactions are younger and less complex. Furthermore, the opposing mechanism of ecological limits to speciation does not apply to young oceanic islands, because diversity is not yet limited by resources as is often the case for mature continental ecosystems^58^.

After establishment of species populations on islands, vicariant differentiation is supported by geological and geomorphological processes^59^, creating heterogeneous site conditions and ecosystems, respectively. The environmental and landscape context of eco-evolutionary processes, however, is rarely addressed^60^, in comparison with spatial constraints and time scales related to phylogenetic molecular mechanisms.

**Fig. 3:**
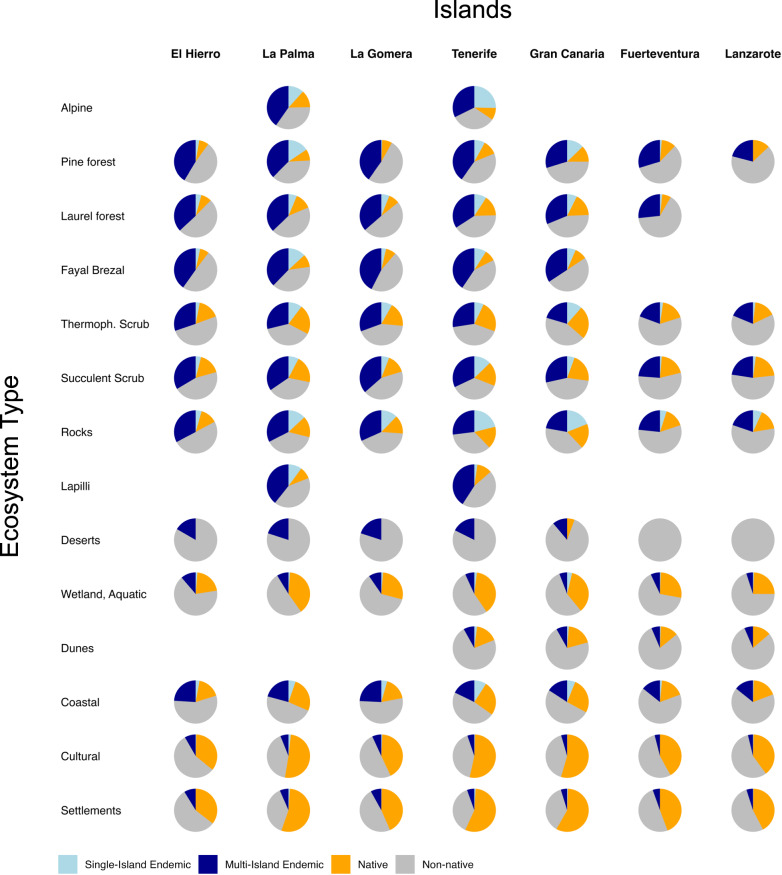
Proportion of Single Island Endemic (SIE), Multi Island Endemic (MIE), native, and non-native plant species in major ecosystems of the Canary Islands. Woody ecosystems such as alpine, pine forest, laurel forest, fayal-brezal, thermophilous scrub, and succulent scrub as well as rocks and lapilli (tephra) sites are dominated by endemic and native plant species on all islands. Deserts, wetlands, and dunes are low in area and species, but proportions are consistent all over the islands with substantial numbers of non-natives in dunes and wetlands. Coastal habitats show this, too, but still there are many MIE species. Cultural land and settlements are dominated by non-native species.

At the level of phytosociological communities, it has been shown that diversified lineages dominate cover on the Canary Islands in only two ecosystems (rocks and summit scrub)^61^. However, ecological success in dominance does not inform about evolutionary success in speciation. In fact, rocks and summit scrub show the highest proportion of Single-Island Endemic plants, but ecosystems that are dominated and structured by less diversified lineages such as genera with only one multi-island endemic (MIE) species (pine forest, laurel forest, fayal brezal), can also show a high proportion of endemism (Fig. 3). It is rather a question if an ecosystem can provide a certain long-term stability of site conditions necessary for the maintenance of endemic species populations as is the case for woody ecosystems in general. If then less competitive pressure for resources, such as light, is combined with relatively short life cycles and population turnover (as is the case in open woodlands, rocks, and summit vegetation), there are windows of opportunity for speciation. Shady forest ecosystems exhibit more long-lived Macaronesian or Multi-Island Endemics while open shrublands exhibit a larger proportion of Single-Island Endemics. In contrast to the dense forests, non-native species can establish there together with native non-endemics.

Remarkably, Canary Island ecosystems differ substantially, ranging from desert-like conditions in the southern lowlands to evergreen laurel forests that thrive on trade-wind clouds and from coastal cliffs to the alpine zone. Only few species range across a large part of these gradients. Most are restricted to only one or a few ecosystems. As described by Species-Area Relationships, ecosystems differ in diversity depending on the area they cover on an island. It is worth asking now whether these principles also apply to endemic plant species. At the global scale, patterns of endemism and Endemics-Area Relationships (EAR) reflecting topography and climate have been detected^62^.

For oceanic islands, where dispersal filters result in lower species richness, SAR are likely to be steeper compared to mainland habitats. Small islands are less likely to be reached and populations are less likely to stay established. This may apply to Endemic-Area Relationships at the scale of entire islands, but also at the scale of ecosystems within islands. With this in mind, it is astonishing that the spatial extent of ecosystems has since now been linked to speciation in only very few case studies (e.g. Yamasaki et al.^63^ for migratory freshwater fish).

**Fig. 4:**
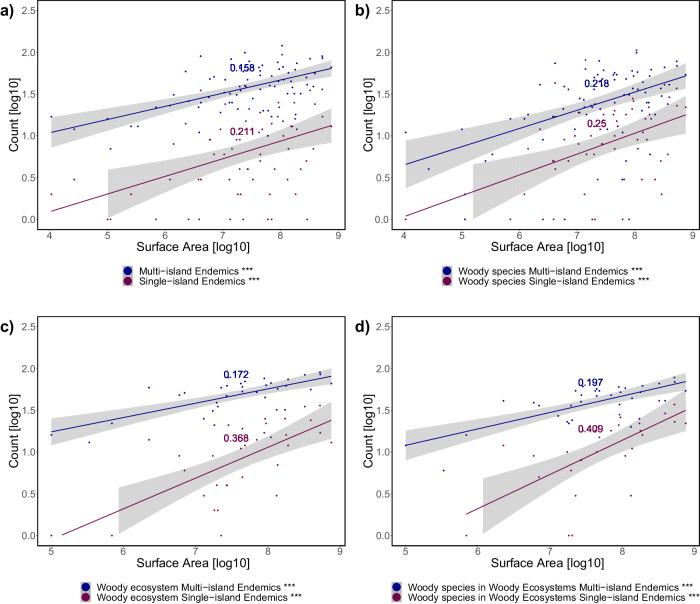
Species Area Relationships (SAR) for Canary Island endemic plants related to the 3D surface area of individual ecosystems from where they have been reported for all islands. Generalized Linear Regression Model (XY logarithmic transformation), Slope information in graph. With increasing surface area of individual ecosystems, the number of endemic species also increases. Single Island Endemics (SIE) show stronger response compared to Multi Island Endemics (MIE) in all cases and woody species as well as woody ecosystems exhibit stronger correlation than all species and all ecosystems. **a** For all endemics; **b** woody endemics; **c** for woody ecosystems only (laurel forest, pine forest, fayal brezal, succulent scrub, thermophilous scrub, alpine scrub); **d** woody species in woody ecosystems.

For the flora of the Canary Islands and their distribution across ecosystems, there is a clear trend of increasing numbers of endemic species (as a proxy for speciation) with an increasing surface area of individual ecosystems within the islands (Fig. 4). Here, Endemics-Area Relationships (EAR) are detected in relation to the real three-dimensional surface that is available for plants. This approach was chosen because of the pronounced topography of most islands. Two dimensional spatial projections can underestimate the available habitat for ecosystems of steep slopes, such as natural forests that have not been subject to strong anthropogenic interferences. An increasing richness of endemic plant taxa applies to both, multi-island endemics (MIE), with populations on several islands of the archipelago, and single-island endemics (SIE), that are restricted to one island. As MIE occur on more than one island, they provide a high number of counts when island endemism is related to the area of specific ecosystems for every island.

The trend towards increasing endemism with surface area is enhanced for woody species. Furthermore, it is strongest for woody ecosystems. Long-lived perennial woody endemic species in permanent woody ecosystems such as forests, woodland, or shrublands have been exposed to biotic interactions over evolutionary time scales. Most of the endemic plant species of the Canary Islands are woody, with a clear dominance of shrubs^49^. Out of 565 shrub species, 258 are SIE and 116 are MIE. The fact that it can be shown that the number of endemic plant species increases with the surface area of ecosystems where they are recorded is evidence for the contribution of ecosystem size to speciation. Once evolved, large ecosystems are more likely to maintain species’ populations.

## Macroecological global vs individualistic local patterns?

Biogeographical patterns in speciation emerge at different scales ranging from entire continents to individual islands as well as from biomes to ecosystems. Long-term climatic conditions and the large-scale spatial arrangement of land surfaces are likely to be reflected in macroecological patterns of speciation and endemism. In contrast, local biotic interactions as well as short-term stochastic processes, such as disturbances, are more likely to result in individualistic and non-deterministic repercussions in local communities and ecosystems^64,65^.

The processes that are supporting or suppressing speciation across the heterogeneous vegetation of islands are manifold. However, species assemblages of endemic plants in ecosystems are not random. They reflect the variety of opportunities for different strategies and life cycles, which are dependent on abiotic site conditions and biotic interactions within ecosystems. This is illustrated by the diversity of phylogenetic ages of endemic plants as well as by mixtures of anagenetic and cladogenetic endemics in different communities. Island syndromes of endemic plant species, such as insular woodiness, may result not only from large-scale and long-term climatic conditions as well as spatial isolation^66,67^, but also from local disturbance regimes such as volcanic eruptions and ash deposition^49^.

In contrast to the etymological message of macroecology (ecology at a macro-scale), this field has until now focused mainly on species lists and their linkages with climate, topography, and earth history. With awareness of the restrictions in knowledge about microclimates of ecosystems and the dependence of soil microorganisms for nutrient uptake, even more so of the influence of community assemblages and biotic interactions during speciation, this crucial aspect to understanding biogeographical patterns should no longer be restricted to single ecological and evolutionary case studies.

## Research perspectives

Future research on endemic plants on oceanic islands should consider, in addition to an island’s total area or spatial isolation, increasingly the community matrix and area of habitat that is provided within individual islands. Islands differ strongly not only in size and elevation but also in the pattern of ecosystems that serve as habitat for plant populations. Then, not the entire island size or elevation per se is relevant for speciation but the spatial arrangement of ecological conditions that can come along with several isolated populations within an island. Furthermore, in addition to abiotic site conditions such as elevation, biotic interactions and biodiversity within communities should be more considered in evolutionary studies.

## Data Availability

No datasets were generated or analysed during the current study.
